# Effects of Raw Materials and Pyrolysis Temperatures on Physicochemical Properties of Biochars Derived from Hemp Stalks

**DOI:** 10.3390/plants14162564

**Published:** 2025-08-18

**Authors:** Xia An, Ziyi Zhu, Xiahong Luo, Changli Chen, Tingting Liu, Lina Zou, Shaocui Li, Yuxue Liu

**Affiliations:** 1 State Key Laboratory for Quality and Safety of Agro-Products, Zhejiang Xiaoshan Institute of Cotton & Bast Fiber Crops, Zhejiang Institute of Landscape Plants and Flowers, Zhejiang Academy of Agricultural Sciences, Hangzhou 311251, China; 13456319193@163.com (Z.Z.); luoxh@zaas.ac.cn (X.L.); chenchangli@zaas.ac.cn (C.C.); liutt@zaas.ac.cn (T.L.); zoulina1991@yeah.net (L.Z.); lishaocui@zaas.ac.cn (S.L.); 2 College of Environment and Resources, College of Carbon Neutrality, Zhejiang A&F University, Hangzhou 311300, China; 3 State Key Laboratory for Quality and Safety of Agro-Products, Institute of Environment Resource Soil and Fertilizer, Zhejiang Academy of Agricultural Sciences, Hangzhou 310021, China

**Keywords:** hemp stalk, biochar, pyrolysis temperature, soil amendment, surface functional group

## Abstract

Hemp stalk, a widely available agricultural waste, is an ideal eco-friendly raw material for biochar production. Carbonization experiments were conducted as a novel approach for the scalable and value-added utilization of hemp stalk under oxygen-exclusion conditions. The effects of feedstock types—*Hibiscus cannabinus* (KS), *Corchorus* spp. (JS), and *Boehmeria* spp. (RS)—and pyrolysis temperatures on biochar properties were analyzed through the measurements of X-ray diffraction (XRD), Fourier transform infrared spectroscopy (FTIR), and X-ray photoelectron spectroscopy. The pH and electrical conductivity (EC) of biochars increased with increasing pyrolysis temperature. Notably, EC was significantly higher for RS (940–2278 μS/cm) than for KS (517–879 μS/cm) and JS (583–863 μS/cm). The C content in these three biochars increased as the temperature increased, whereas the H/C atomic ratio decreased, most notably in JS (by 0.33%). According to FTIR and XRD data, with the pyrolysis temperature increasing, the acidic oxygen-containing groups on biochar surfaces reduced. KS700, with superior aromatic structure and stability, may be able to effectively adsorb heavy metal ions. RS700, with relatively high pH and EC, was suitable for alleviating soil acidification and nutrient deficiency. The feedstock and pyrolysis temperature significantly affected the element content, pore structure, and stability of biochars derived from hemp stalk.

## 1. Introduction

Population growth has been accompanied by increases in resource consumption, urban expansion, energy demands, and ecological imbalances, which have severely affected the environment. A significant challenge in China, a major agricultural country, is managing the large volume of biomass waste generated during crop harvest. Specifically, the treatment of biomass waste has become the main bottleneck restricting sustainable agricultural development. Among them, hemp straw, as a common agricultural waste, has a considerable output, but the current treatment methods are mostly crude, often disposed of through incineration, disposal, and other means. This not only causes huge waste of resources but also may lead to a series of negative problems, such as environmental pollution. According to FAO data, the global hemp planting area in 2019 was approximately 546.2 million hectares, with a yield of 2.8 million tons. China is the world’s largest producer of hemp, with a planting area of over two million hectares and a yield accounting for 40% of the global total. Asian countries such as India, Brazil, and Bangladesh also hold significant market shares [1]. Hemp, which is characterized by a short growth cycle, high biomass and energy yield, and strong adaptability, can be grown on marginal land, ensuring a stable and sustainable raw material supply without competing with food crops [2]. Hemp stalk, a prevalent agricultural waste rich in cellulose and lignin [3], undergoes efficient C conversion during pyrolysis. The obtained biochar, with its well-developed pore structure and large specific surface area, exhibits excellent adsorption performance and soil improvement potential. Moreover, it can effectively enhance water–fertilizer retention and C sequestration in soils [4,5]. In addition, using hemp stalk for biochar production recycles agricultural waste, mitigates pollution from open burning, and advances the circular economy in agriculture, offering a viable technical route towards C neutrality [6,7,8].

There is growing interest in the utility of biochar as an eco-friendly material, thereby necessitating ongoing research [9,10,11]. Biochar, a carbon (C)-rich material derived from renewable biomass via thermochemical conversion, resembles natural products and has diverse uses (e.g., environmental remediation and agricultural production) [12,13]. Its formation typically involves the pyrolysis or gasification of biomass under oxygen-limited conditions, ultimately resulting in highly stable solid C products [14]. Earlier research showed that biochar durably sequesters C and decreases greenhouse gas (e.g., CO_2_, CH_4_, and N_2_O) emissions [15,16,17]. Biochar can also serve as an electron acceptor, enhancing the photocatalytic ability of catalysts [18,19]. Additionally, it improves the soil structure and enhances nutrient retention, thereby increasing the efficiency of crop nutrient utilization. Moreover, its high calorific value and renewability make biochar a sustainable alternative to fossil fuels, with potential significant implications for energy and resource recycling [15,20,21].

Biochars from various sources differ significantly in terms of physical, chemical, and biological properties because of the diversity in their raw materials, pyrolysis parameters (e.g., temperature, heating rate, and residence time), and post-treatment changes (e.g., activation and modification). Hence, depending on their source, biochars may vary regarding their effects and potential utility in various fields (e.g., agriculture, environmental remediation, and energy). Sørmo et al. determined that both sludge-based and activated wood biochars are suitable for the immobilization and adsorption of PFAS in contaminated soils [22]. Sabatino et al. reported that peat-modified biochar affects the yield, nutrition, and functional traits of chicory, depending on the biochar percentage and pyrolysis temperature [23]. Fernandez et al. identified walnut shell-based biochar with excellent biofuel potential because of its high energy density and low ash content [24]. Musa et al. observed that biochar derived from manure (including farmyard manure and poultry manure) adsorbed more phosphorus at 400 °C than at 600 °C [25].

At present, there are few reports on the study of the interaction between the physical and chemical properties of biochar and the dual factors of raw materials themselves and pyrolysis temperature, and the exploration of the optimal carbonization process conditions in practical applications such as soil improvement and environmental remediation [26,27,28]. In particular, there have been few feasibility studies on using hemp stalks (instead of traditional crop straws) as raw materials to prepare biochar in a systematic manner. Based on this blank, this study uses kenaf, jute, and ramie as raw materials to prepare biochar by controlling different pyrolysis temperatures. The research aims to open up an efficient and environmentally friendly new path for the utilization of cannabis straw resources, transforming them into soil improvement and environmental remediation materials with broad application prospects. At the same time, we hope to provide important theoretical support and technical references for promoting the sustainable development of agricultural waste and ecological environment protection. At the same time, it is also expected to inject new vitality and innovative ideas into the research of the biochar field, further expand the application scope and depth of biochar technology in different fields, and achieve a win–win situation of economic and environmental benefits.

## 2. Materials and Methods

### 2.1. Experimental Materials

In the present study, we used hemp stalks from three different sources, namely *Hibiscus cannabinus* (KS), *Corchorus* spp. (JS), and *Boehmeria* spp. (RS), as raw materials to prepare biochar at pyrolysis temperatures of 300, 500, and 700 °C. KS, JS, and RS samples were sourced from a hemp breeding base (30°4′13″ N, 120°13′34″ E; 7.95 m elevation) in Hangzhou, Zhejiang Province, China. More specifically, the collected samples were xylem fractions, with bast fiber removed, from mature hemp fibers. For the collection and pre-treatment of samples, hemp plants with mature fibers were uprooted, after which soil and stem branches were manually removed. After the roots were completely separated from the stem, the bast fibers were stripped, and the xylem was retained. Treated hemp stalks (i.e., xylem with bast fibers removed) were rinsed with deionized water and then air-dried in a cool, ventilated area for 12 h to eliminate excess moisture. Samples were dried to a constant weight in a 70 °C oven for more than 24 h.

### 2.2. Preparation of Biochars

**Figure 1:**
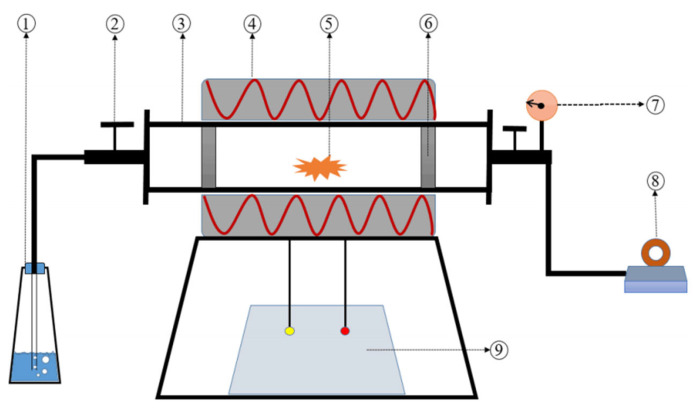
Schematic diagram of the pyrolysis setup. (1) Exhaust gas absorber; (2) valve; (3) quartz tube; (4) heating jacket; (5) biomass; (6) filter plug; (7) pressure meter; (8) vacuum pump; (9) temperature controller [29].

**Figure 2:**
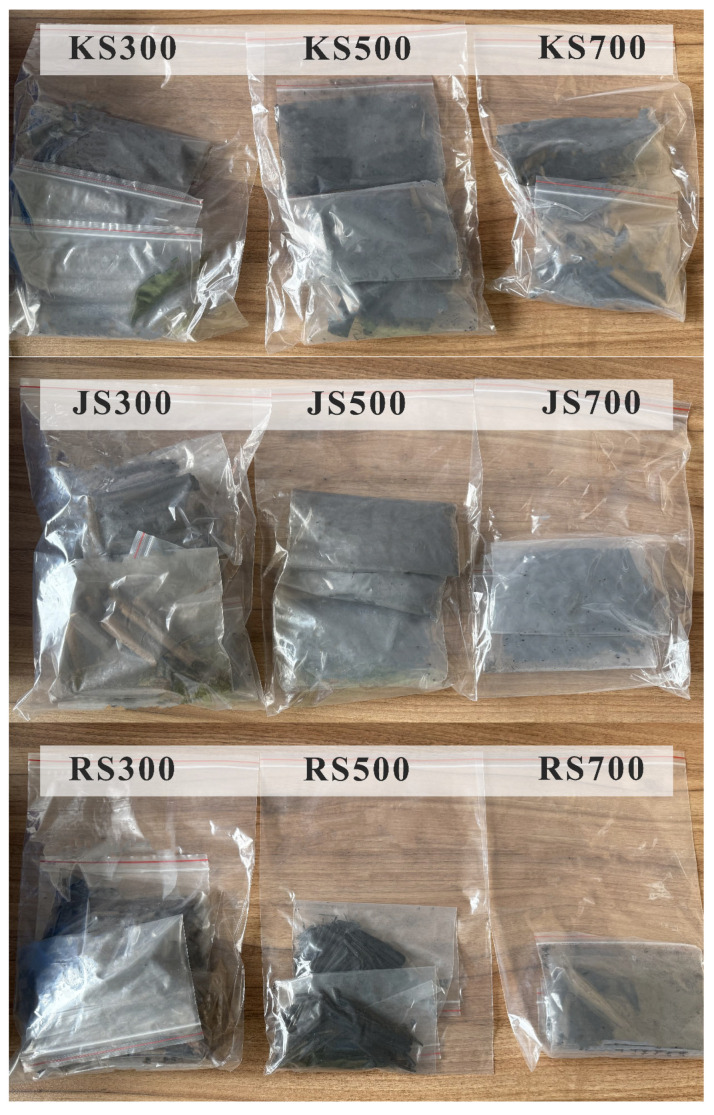
Nine samples of biochar.

A tube furnace (Hangzhou Lantian Instrument Co., Ltd., Hangzhou, China, Figure 1) was used for the slow pyrolysis of biomass materials in a strictly controlled anaerobic environment. The diameter of the instrument is 100 mm, and the tube is placed horizontally for heating. The starting temperature of the tubular electric furnace is set to 30 °C, the heating rate is set to 20 °C/min, and the temperatures are raised to 300, 500, and 700 °C, respectively, and kept at constant temperature for 2 h. Biochar samples were naturally cooled to room temperature after reaching the target temperature (Figure 2). This process ensured the hemp stalk-derived biochars met the quality and performance standards required for this study.

Finally, samples were ground and passed through a 100-mesh sieve. The filtered ground material was kept in a sealed plastic bag until analyzed. For convenience, according to their source and pyrolysis temperature, samples were designated as KS300, KS500, and KS700 (kenaf stalk-derived biochar), JS300, JS500, and JS700 (jute stalk-derived biochar), and RS300, RS500, and RS700 (ramie stalk-derived biochar). All biochar samples were ground to a fine powder using a ball mill, dried overnight at 105 °C, and then analyzed.

### 2.3. Experimental Methods

At a 1:20 (*w*/*v*) dilution, the physicochemical properties of KS, JS, and RS were thoroughly investigated. Each suspension was shaken and then left undisturbed for 5 min to stabilize before pH was measured. Subsequently, electrical conductivity (EC) was determined. A Vario EL/micro cube (Elementar, Langenselbold, Germany) was used to measure the nitrogen (N), C, hydrogen (H), and sulfur (S) contents in the three biochars, thereby clarifying their chemical composition.

Inorganic components were identified via X-ray diffraction (XRD) using a diffractometer (D8 Advance, Bruker, Bremen, Germany) at a scan rate of 0.01 s^−1^ and an angular range of 0–90° within 2 h. Fourier transform infrared spectroscopy (FTIR) (400–4000 cm^−1^; Nexus 6700, Thermo Fisher Scientific, Waltham, MA, USA) and X-ray photoelectron spectroscopy (XPS) (ESCALAB 250Xi, Thermo Fisher Scientific, Shanghai, China) analyses were conducted to characterize the changes in functional groups, major elements, and chemical states of hemp stalk-derived biochars at room temperature.

### 2.4. Data Analysis

Statistical analyses were completed using Excel 2020. Jade 9 was used to analyze XRD data, whereas Origin 2024 was used to analyze FTIR spectra. Avantage 6.6 was used for the fitting of XPS data peaks. Origin 2024 was used for graphing.

## 3. Results

### 3.1. Basic Physicochemical Properties of Biochars

Table 1 presents the physicochemical properties of three hemp stalk-derived biochars at varying temperatures (i.e., pH; EC; N, C, H, and S contents; and atomic ratios). Table 2 shows the yield of nine parts of hemp stem biochar. The yield of biochar decreases with increasing temperature. According to Table 3, the equilibrium moisture content of jute stem biochar is higher than the other two, and the equilibrium moisture content of JS300 reaches 20.3%. In addition, the bulk density of the three types of biochar is similar, with jute stem biochar having a lower density compared to the other two, and JS500 having the lowest density of 0.029 g/cm^3^. Temperature increases resulted in increases in pH from weakly acidic to alkaline, likely because of decreases in organic matter contents and inorganic ion binding in the raw materials. At 300–700 °C, pH increased from 6.60 to 9.23 for KS, 6.23 to 9.01 for JS, and 6.89 to 9.32 for RS. At the same pyrolysis temperature, RS had the highest pH, followed by KS and then JS. Additionally, under the same conditions, EC increased for KS, JS, and RS as the pyrolysis temperature increased, but EC was significantly higher for RS (940–2278 μS/cm) than for KS (517–879 μS/cm) and JS (583–863 μS/cm).

As the pyrolysis temperature increased, the C content increased from 66.8% to 82.8% in KS, 51.7% to 81.2% in JS, and 48.8% to 74.6% in RS. The increase in the C content was greatest for JS (57.1%), followed by RS (53.1%) and KS (24.0%). At the same temperature, KS had the highest C content. The rank order of the three samples in terms of their C content was maintained at KS > JS > RS as the pyrolysis temperature increased. The S content in KS and JS initially increased and then decreased, similar to the N content in JS and RS. However, both the H content in the three biochars and the N content in KS increased as the temperature increased. At the same temperature, the S content was highest in JS, followed by KS and RS. Additionally, element contents varied significantly among biochars produced from different raw materials and at different pyrolysis temperatures. More specifically, the N content was highest in RS500 (0.87%) and lowest in JS300 (0.29%); the H content was highest in KS700 (4.09%) and lowest in RS300 (3.78%); and the S content was highest in JS500 (0.58%) and lowest in RS300 (0.13%). Because of the low N and S contents in hemp stalks, the derived biochars were highly stable and eco-friendly, with minimal NO_x_/SO_2_ emissions.

Under the same conditions, the H:C ratio of the three biochars decreased as the temperature increased, likely because of increases in biochar aromaticity during pyrolysis [30]. At 300–700 °C, H:C ratios were 0.59–0.69 for KS, 0.60–0.89 for JS, and 0.63–0.93 for RS. The decrease in the H:C ratio was largest for JS (0.33%), followed by RS (0.32%) and KS (0.14%). Additionally, at the same temperature, RS and KS had the highest and lowest H:C ratios, respectively. By contrast, C:N ratios were high for all three biochars (KS, 227.6–258.6; JS, 197.3–232.6; and RS, 90.7–135.9). The C:N ratio was highest for KS500 (258.6) and lowest for RS500 (90.7). Earlier research showed that biomass with a high C:N ratio can yield biochars that can effectively sequester C [31].

### 3.2. XRD Patterns of Biochars

**Figure 3:**
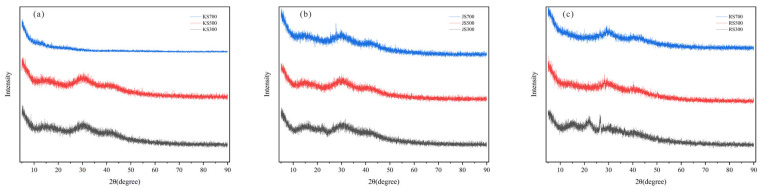
XRD diffraction patterns of biochar. (**a**) KS, (**b**) JS, and (**c**) RS. The black line represents the biochar sample with a pyrolysis temperature of 300 °C; The red line represents the biochar sample with a pyrolysis temperature of 500 °C; The blue line represents the biochar sample with a pyrolysis temperature of 700 °C.

Figure 3 depicts the XRD patterns of KS, JS, and RS at various pyrolysis temperatures. XRD data are useful for clarifying the structure and mineral composition of biochar. With the exception of KS700, all samples had a consistent, significant C peak near 30° (Figure 3), with peak sharpness and intensity largely unaffected by the pyrolysis temperature. Thus, all of these samples were C materials with typical local arrangements.

Increases in the pyrolysis temperature were accompanied by the release of volatile components from the biomass, resulting in the structural reorganization of the C-based materials and the formation of new biochar structures [32]. Quartz (SiO_2_, 2θ ≈ 24°) was found in KS, JS, and RS at 300 °C, whereas potassium salt (KCl, 2θ ≈ 28°) was present in KS and RS at 500 °C and calcite (CaCO_3_, 2θ ≈ 27°) was detected in JS and RS at 300 °C. Hydrated lime [Ca(OH)_2_, 2θ ≈ 32°] was detectable in RS700.

### 3.3. FTIR

**Figure 4:**
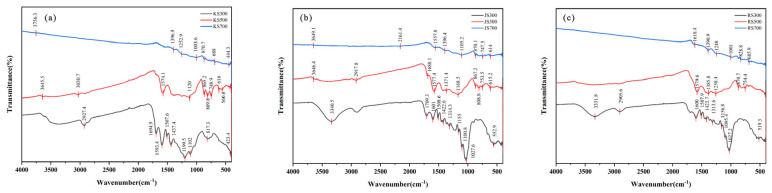
FTIR spectra of biochar. (**a**) KS, (**b**) JS, and (**c**) RS.

Figure 4 shows the functional groups of KS, JS, and RS in FTIR spectra. As the temperature increased, large or small peaks for all three biochars flattened and some even disappeared, reflecting the significant effect of the pyrolysis temperature on the number and types of functional groups on the biochar surface. At 300 °C, both JS and RS had prominent adsorption peaks at 1600, 1507.9, 1422.6, 1314.3, 1156.8, and 1027.6 cm^−1^, with similar characteristic adsorption peaks, suggesting that these two biochars had highly similar or identical functional groups. By contrast, KS had distinct peaks (in terms of shape and number) between 1750 and 1010 cm^−1^. At 500 °C, both KS and JS had similar peaks at 3646.5, 1577.4, 1168.5, 865.2, 808.8, and 615.2 cm^−1^, implying that the surface of these two biochars had more functional groups and a higher adsorption capacity than the surface of RS, which had fewer peaks. Thus, under identical conditions, the biochars from different raw materials differed significantly in terms of their functional groups.

As the temperature increased, KS peaks decreased, with some peaks disappearing at 700 °C (i.e., at 3645.5, 3030.7, 1574.1, 1003.6, 817.3, 616, and 4423.4 cm^−1^). Similarly, JS peaks also decreased as the temperature increased (i.e., at 2917.8, 1709.3, 1508.6, 1314.3, and 1027.6 cm^−1^). RS peaks were present at 300 °C but were undetectable at 500 and 700 °C (i.e., at 3331.8, 2905.6, 1507.9, 1156.8, and 519.3 cm^−1^). At 500 and 700 °C, KS, JS, and RS had dense peaks (at 560.6–865.2 cm^−1^) that were absent at 300 °C. At 300 °C, KS, JS, and RS had strong and broad adsorption peaks at 3000–3500 cm^−1^, reflecting the presence of a hydroxyl (-OH) group. These peaks were mainly the adsorption and stretching vibration peaks of the carboxyl group (-COOH) or hydroxyl groups in water molecules. The peak at 2905 cm^−1^, which likely represented CH_2_ stretching, disappeared at 700 °C. Furthermore, the peak near 1605 cm^−1^ was possibly due to the C=O stretching vibration in carboxyl groups.

### 3.4. XPS

**Figure 5:**
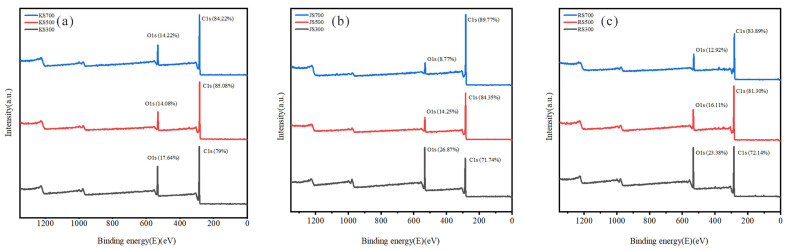
Full XPS spectra of biochar. (**a**) KS, (**b**) JS, and (**c**) RS.

As indicated in Figure 5, C and O were the main elements at the surface of the nine biochars. As the pyrolysis temperature increased, the C1s peak intensity for the surface functional groups of JS and RS biochars increased significantly, which was in contrast to the decrease in the O1s peak intensity, indicating that the biochars gradually decomposed as the pyrolysis temperature increased, while their structures stabilized. At 700 °C, the C1s peak intensity of KS decreased below the level at 500 °C, possibly because new structures formed at high temperatures, thereby affecting the C1s signal intensity.

**Figure 6:**
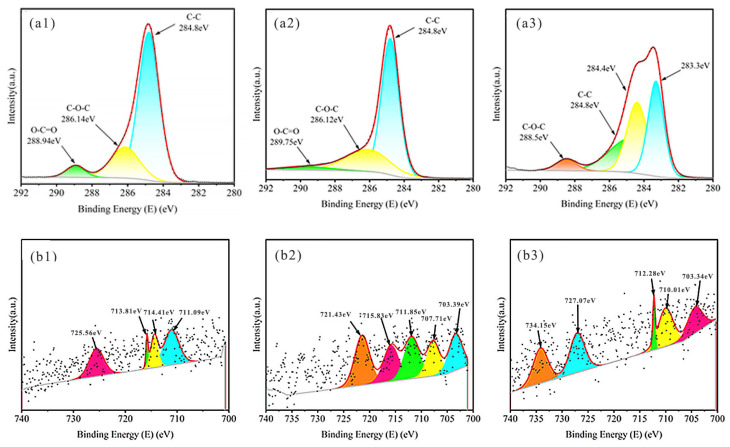
XPS spectra of KS. XPS spectra of C1s for (**a1**–**a3**) KS300, KS500, KS700; XPS spectra of Fe2p for (**b1**–**b3**) KS300, KS500, KS700. Colored curves are peak—fitting results, representing the deconvoluted chemical states of elements; Black dots are raw data points from XPS measurement, showing the original signal intensity—binding energy relationship.

**Figure 7:**
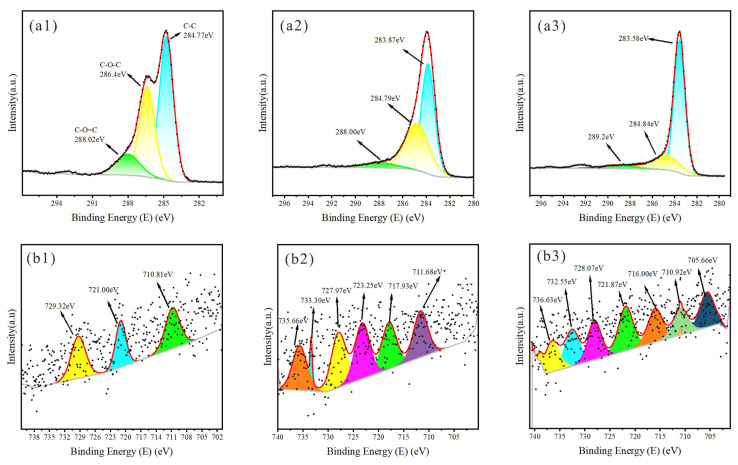
XPS spectra of JS. XPS spectra of C1s for (**a1**–**a3**) JS300, JS500, JS700; XPS spectra of Fe2p for (**b1**–**b3**) JS300, JS500, JS700; Colored curves are peak-fitting results, representing the deconvoluted chemical states of elements; Black dots are raw data points from XPS measurement, showing the original signal intensity-binding energy relationship.

**Figure 8:**
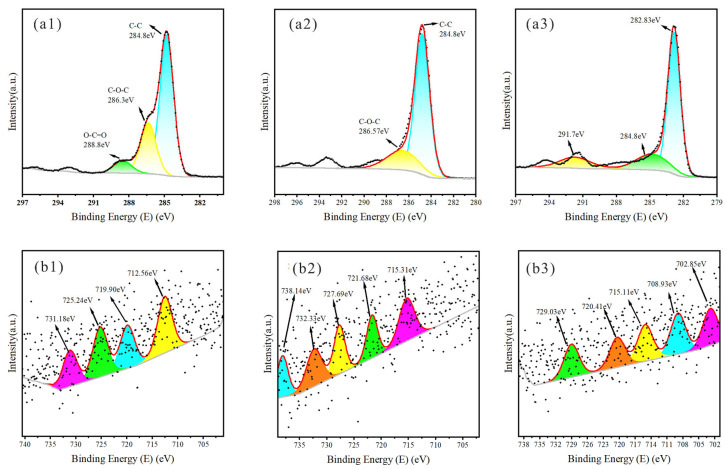
XPS spectra of RS. XPS spectra of C1s for (**a1**–**a3**) RS300, RS500, RS700; XPS spectra of Fe2p for (**b1**–**b3**) RS300, RS500, RS700; Colored curves are peak—fitting results, representing the deconvoluted chemical states of elements; Black dots are raw data points from XPS measurement, showing the original signal intensity—binding energy relationship.

Figure 6, Figure 7 and Figure 8 present XPS spectra for KS, JS, and RS, respectively. At 300 and 500 °C, most of the C peaks for all three biochars were near 284.8 eV, suggestive of a hydrocarbon-rich surface with a dominant C skeleton. As the pyrolysis temperature increased, the shapes of C peaks changed markedly, signifying more substantial structural changes in C, with additional organic functional groups transformed or lost, leading to the formation of relatively stable graphitized C. The C-O-C peak near 286.14 eV indicated the presence of ethers or alcohols formed by C and oxygen, suggesting that there were still many oxidizing functional groups on the biochar surface. As the temperature increased, the peak was significantly suppressed, reflecting the near-complete removal of oxygen-containing functional groups at high temperatures, with a predominantly graphitic C skeleton remaining.

Iron predominantly existed as Fe^3+^ near peaks at 711.09 and 714.41 eV, but other forms were possible, including Fe_2_O_3_ or other oxygenated compounds. Temperature increases may result in the formation of metallic iron or low-valence iron oxide. Iron oxides were present near peaks at 727.07 and 734.15 eV at 700 °C.

## 4. Discussion

Thermochemical biomass conversion may involve dehydration, decarboxylation, polymerization, and aromatization [33]. By characterizing the physicochemical properties of three hemp stalk-derived biochars at 300, 500, and 700 °C, we determined that the pyrolysis temperature and raw material type significantly affected biochar properties and structures, which is in accordance with the findings of earlier research [29,34,35].

### 4.1. Effects of Pyrolysis Temperatures on Hemp Stalk-Derived Biochars

The pyrolysis temperature significantly influences the number and types of functional groups at the biochar surface, while also affecting the ability of biochar to adsorb heavy metal ions from aqueous solutions [36,37]. At relatively low preparation temperatures, biochar surfaces are rich in organic anionic oxygen-containing groups (e.g., -COO- and -O-), which produce acidic substances that partially reside in the biochar [38]. Increases in the pyrolysis temperature facilitate the volatilization of acidic substances and the precipitation of alkali metals [39]. In the present study, we observed that high pyrolysis temperatures increased the pH and EC of hemp stalk-derived biochars (from weakly acidic to alkaline). The increased alkalinity positively affected the specific surface area and introduced oxygen-containing functional groups, which is consistent with the results of previous research.

Increasing the pyrolysis temperature can increase the C content of biochar [40]. The C content of biochar is mostly between 30% and 90%, with a high C content being a key attribute of biochar suitable for soil amendments [41]. By contrast, a low H:C ratio indicates high aromaticity and stability; biochars with a highly aromatic structure have high antioxidant and adsorption capacities. Mixed kitchen waste-derived biochars have a C content of 48–55% [42], rice straw-derived biochars have a C:N ratio of 56.9–71.5% [29], and corn straw- and *Miscanthus*-derived biochars have a C content of 42–47% [43]. Increasing the pyrolysis temperature increases the specific surface area of soybean straw-derived biochar, significantly improving its Cd^2+^-adsorbing capacity. Biochars prepared at 600 °C have the highest adsorption capacity (31.98 mg/g), with the adsorption peak of the -OH group appearing at 3400–3420 cm^−1^ along with the stretching peak of the aromatic carbonyl/carboxyl (C–O) group at 1600 cm^−1^. Although the peak intensities of the -OH and C–O groups decrease at high temperatures, these groups remain crucial for Cd^2+^ adsorption [44]. The yield of sugarcane-derived biochar decreases by 71.6% as the temperature increases from 400 to 800 °C, but the S content increases [45]. In the present study, the analyzed hemp stalk-derived biochars had a high C:N ratio, especially KS500 (258.60%), reflecting the high C and low N contents in these biochars. The H:C ratio of hemp stalk-derived biochars decreased as the pyrolysis temperature increased, with KS having the lowest H:C ratio (i.e., optimal stability).

Increasing pyrolysis temperatures can enhance biochar aromatization [31]. In the current study, XRD spectra revealed a distinct C peak at 700 °C for all three biochars, with peak sharpness and intensity remaining relatively constant across temperatures. Moreover, all three hemp stalk-derived biochars were rich in oxygen-containing functional groups with a similar thermal series. Biochar carboxyl groups, which are bound by hydrogen bonds, break during pyrolysis, and the -OH vibration peaks (3000–3500 cm^−1^) in all three biochars decreased as the temperature increased, becoming undetectable at 700 °C. At 2800–3000 cm^−1^, aliphatic CH_3_ and CH_2_ groups emerged (possibly because of decarboxylation), but they were absent at 700 °C, indicating a shift from aliphatic to aromatic biochars [46]. The O1s peak intensity of KS decreased as the pyrolysis temperature increased, indicative of anaerobic combustion. Conversely, C1s peak intensities of JS and RS increased significantly as the pyrolysis temperature increased, which is consistent with reported research findings. At 300 and 500 °C, C skeletons were abundant at the biochar surface; however, increases in the pyrolysis temperature resulted in a loss of organic groups and the formation of relatively stable graphitized C.

### 4.2. Effects of Raw Materials on Hemp Stalk-Derived Biochar Physicochemical Properties

Raw materials significantly affect biochar physicochemical properties [47]. Accordingly, raw materials influence the quality, potential applications, and elemental composition (including fixed C and ash content) of biochars [48]. Previous research showed that the N content in raw materials directly influences the N content in biochars. For example, biochars produced from soybean straw, which contains 2.21% N, have an N content of 1.79–3.49%. By contrast, the N content of *Chlorella* microalgae (10.80% N)-based biochars ranges from 7.16% to 11.34% [47]. Otoni et al. determined that wood chip- and bagasse-based biochars have the highest C content, followed by fruit shell- and grass chip-based biochars, with poultry manure-derived biochar having the lowest C content [31]. Wood-derived biochar has a relatively high C content; however, manure- or crop residue-derived biochars are better for increasing soil microbial abundance than biochars produced from wood or other raw materials rich in lignocellulose [49]. In another study, biochar produced from the annual shoots of holly oak had higher lignin (23.60%) and calcium (2.64%) contents than a vine-based biochar (20.16% lignin and 1.13% calcium), resulting in greater mechanical strength and impact resistance [50]. Pineapple leaf- and banana stem-derived biochars, with high pH and abundant alkaline minerals, are suitable for the amendment of acidic soils. Bagasse- and horticultural substrate-based biochars have high C contents and large specific surface areas, which are conducive to promoting plant growth [51].

In the present study, raw materials had relatively minor effects on biochar pH. At the same temperature, RS had the highest pH, and its EC was significantly higher than that of the other samples, indicating biochar EC is significantly influenced by the raw material. Additionally, biochars from different raw materials differed significantly regarding elemental contents, with KS having the highest C content. However, the C content increased the most in JS (57.1%), consistent with the XPS spectra.

Wood-derived biochar weakly decreases the soil N content, whereas biochars produced from crop residues (e.g., corn stalks) or carbohydrate materials (e.g., husk) have more notable effects [52]. For example, soybean straw-derived biochar, with large pores (1.17 nm) and abundant hydroxyl/carboxyl groups, can efficiently remove antibiotics (e.g., norfloxacin) from wastewater [53]. Activated wood waste- and sludge-based biochars, with high specific surface areas and large pores (>1.5 nm), can effectively decrease the leaching of perfluorooctane sulfonate [22]. Additionally, activated wood waste-derived biochar treated with chromated copper arsenate has excellent electrochemical properties, with a specific capacitance of 76.7 F/g [54]. In the present study, all three hemp stalk-derived biochars had a typical local ordered C structure. KS, with its high C content that increases with temperature increases, may remain in soil for long periods and sequester C. It also had a low H:C ratio, indicative of a highly aromatic internal structure and enhanced chemical stability. Because of the abundant active sites and relatively large surface area due to its aromatic structure, KS has better adsorption-related properties than the other biochars. The physicochemical properties of JS varied among pyrolysis temperatures, with JS700 superior to JS300. RS had a relatively high pH and EC, and it may be useful for enhancing soil physicochemical properties, increasing the cation exchange capacity, inducing soil aggregate formation, and improving water and fertilizer retention, thereby promoting plant health.

### 4.3. Summary and Prospect

The pyrolysis temperature significantly affects biochar physicochemical properties, and biochars derived from different raw materials have varying physicochemical properties and uses. In addition, biochar has become a promising carbon sequestration material due to its stability [55]. KS is rich in C and has a low H/C ratio, which is thermally stable. The C content of biochar increases with the increase in pyrolysis temperature, and it reaches 82.8% for KS700 sample. This is beneficial for long-term carbon sequestration in soil, thereby reducing the greenhouse effect and improving soil quality. However, the yield of biochar shows a significant decreasing trend with the increase in pyrolysis temperature. Thus, the optimal temperature for carbon sequestration may be between 500 and 600 °C. In addition, its low H:C ratio results in superior chemical stability and heavy metal adsorption and enables the effective immobilization of solid pollutants. By contrast, RS, which is characterized by high pH and EC, is useful for regulating the soil acid–base balance and enhancing cation exchange and water–fertilizer retention. Precisely adjusting the pyrolysis temperature may enable the targeted preparation of biochars for various applications (e.g., C sequestration, emission reduction, pollution remediation, and soil improvement), thus optimizing resource utilization. However, hemp stalk-derived biochars have complex effects on soil and crops, and the effects of combined factors must be quantitatively analyzed in long-term experiments. For example, the water-soluble carbon components in biochar may leach out due to rainfall or irrigation, and physical fragmentation caused by soil cultivation or biological activities may increase its surface area and accelerate degradation; In addition, some soil microorganisms may decompose the activated carbon portion in biochar. Meanwhile, in order to conduct biochar carbon sequestration testing and ensure stability, the following methods can also be used: thermogravimetric analysis (TGA) evaluates thermal stability by monitoring weight loss under oxidative conditions, chemical oxidation tests using potassium permanganate or hydrogen peroxide to measure carbon release, and microbial culture experiments to track changes in biochar carbon content over 6–12 months. In addition, the stability of biochar can be improved by optimizing pyrolysis parameters, coating biochar with polymer or clay materials to reduce contact with water and microorganisms, and applying biochar in deep soil with low microbial activity and water movement to slow down degradation [56].

The present study takes kenaf, jute, and ramie as raw materials for the first time, offering insights into the characteristics of hemp stalk-derived biochars prepared from different raw materials and their potential uses as adsorbents, filter media, and soil enhancers.

Future research priorities may include the following: (1) biochars produced from hemp stalks at various pyrolysis temperatures for N and water retention in soils, with a particular focus on their porous structures and oxygen-containing groups; (2) adsorption mechanisms of hemp stalk-derived biochars for heavy metals and organic pollutants; and (3) potential industrial utility of hemp stalk-derived biochars as catalyst carriers, energy materials, and low-cost electrodes or photocatalytic composites.

## Figures and Tables

**Table 1:** Basic physical and chemical properties of hemp stem biochar from different raw materials at different temperatures ^0^ (*p* < 0.05).

Sample	pH	EC (μS/cm)	C (%)	N (%)	H (%)	S (%)	C/N	H/C
KS300	6.60 ± 0.03 f	517 ± 7.07 f	66.8 ± 0.03 f	0.31 ± 0.01 d	3.85 ± 0.01 c	0.31 ± 0.01 d	249.8	0.69
KS500	7.35 ± 0.04 d	469 ± 8.49 g	79.0 ± 0.09 c	0.36 ± 0.01 cd	4.04 ± 0.08 ab	0.36 ± 0.01 c	258.6	0.61
KS700	9.23 ± 0.04 ab	879 ± 5.66 d	82.8 ± 0.14 a	0.42 ± 0.01 dc	4.09 ± 0.06 a	0.29 ± 0.02 d	227.6	0.59
JS300	6.23 ± 0.03 g	583 ± 10.6 e	51.7 ± 0.20 g	0.29 ± 0.04 d	3.84 ± 0.02 c	0.40 ± 0.02 b	208.5	0.89
JS500	6.67 ± 0.04 f	540 ± 7.78 f	72.6 ± 1.39 e	0.43 ± 0.03 c	3.90 ± 0.07 bc	0.58 ± 0.01 a	197.3	0.64
JS700	9.01 ± 0.06 c	863 ± 16.3 d	81.2 ± 0.09 b	0.41 ± 0.05 c	4.05 ± 0.04 ab	0.57 ± 0.01 a	232.6	0.60
RS300	6.89 ± 0.05 e	940 ± 12.7 c	48.8 ± 0.48 h	0.62 ± 0.01 b	3.78 ± 0.02 c	0.13 ± 0.01 e	91.7	0.93
RS500	9.18 ± 0.06 b	1216 ± 16.3 b	67.5 ± 0.08 f	0.87 ± 0.03 a	3.81 ± 0.09 c	0.15 ± 0.02 e	90.7	0.68
RS700	9.32 ± 0.04 a	2278 ± 9.90 a	74.6 ± 0.14 d	0.64 ± 0.05 b	3.94 ± 0.14 abc	0.15 ± 0.01 e	135.9	0.63

^0^ KS300, KS500, and KS700 (kenaf stalk-derived biochar); JS300, JS500, and JS700 (jute stalk-derived biochar); RS300, RS500, and RS700 (ramie stalk-derived biochar); The same letter indicates no significant difference between groups (*p* > 0.05), while different letters indicate significant differences between groups (*p* < 0.05).

**Table 2:** Production rate of hemp stem biochar.

Sample	Yield (%)
KS300	60.51
KS500	39.60
KS700	24.29
JS300	57.57
JS500	28.25
JS700	23.75
RS300	55.12
RS500	32.37
RS700	24.86

**Table 3:** Bulk density and equilibrium moisture content of biochar.

Sample	Equilibrium Moisture Content (%)	Bulk Density (g/cm^3^)
KS300	5.9	0.058
KS500	3.1	0.059
KS700	7.6	0.042
JS300	20.3	0.046
JS500	15.7	0.029
JS700	10.5	0.035
RS300	8.3	0.055
RS500	6.1	0.056
RS700	8.9	0.095

## Data Availability

Data are contained within the article.
